# Utilization of Random Forest Classifier and Artificial Neural Network for Predicting Factors Influencing the Perceived Usability of COVID-19 Contact Tracing “MorChana” in Thailand

**DOI:** 10.3390/ijerph19137979

**Published:** 2022-06-29

**Authors:** Ardvin Kester S. Ong, Yogi Tri Prasetyo, Nattakit Yuduang, Reny Nadlifatin, Satria Fadil Persada, Kirstien Paola E. Robas, Thanatorn Chuenyindee, Thapanat Buaphiban

**Affiliations:** 1 School of Industrial Engineering and Engineering Management, Mapúa University, 658 Muralla St., Intramuros, Manila 1002, Philippines; aksong@mapua.edu.ph (A.K.S.O.); nuttakit33@gmail.com (N.Y.); kperobas@mymail.mapua.edu.ph (K.P.E.R.); 2 Department of Industrial Engineering and Management, Yuan Ze University, 135 Yuan-Tung Road, Chung-Li 32003, Taiwan; 3 School of Graduate Studies, Mapúa University, 658 Muralla St., Intramuros, Manila 1002, Philippines; 4 Department of Information Systems, Institut Teknologi Sepuluh Nopember, Kampus ITS Sukolilo, Surabaya 60111, Indonesia; reny@its.ac.id; 5 Entrepreneurship Department, BINUS Business School Undergraduate Program, Bina Nusantara University, Jakarta 11480, Indonesia; satria.fadil@binus.ac.id; 6 Department of Industrial Engineering and Aviation Management, Navaminda Kasatriyadhiraj Royal Air Force Academy, Bangkok 10220, Thailand; thanatorn_chu@rtaf.mi.th (T.C.); thapanat_bua@rtaf.mi.th (T.B.)

**Keywords:** contact tracing, artificial neural network, random forest classifier, machine learning algorithm, human behavior

## Abstract

With the constant mutation of COVID-19 variants, the need to reduce the spread should be explored. MorChana is a mobile application utilized in Thailand to help mitigate the spread of the virus. This study aimed to explore factors affecting the actual use (AU) of the application through the use of machine learning algorithms (MLA) such as Random Forest Classifier (RFC) and Artificial Neural Network (ANN). An integrated Protection Motivation Theory (PMT) and the Unified Theory of Acceptance and Use of Technology (UTAUT) were considered. Using convenience sampling, a total of 907 valid responses from those who answered the online survey were voluntarily gathered. With 93.00% and 98.12% accuracy from RFC and ANN, it was seen that hedonic motivation and facilitating conditions were seen to be factors affecting very high AU; while habit and understanding led to high AU. It was seen that when people understand the impact and causes of the COVID-19 pandemic’s aftermath, its severity, and also see a way to reduce it, it would lead to the actual usage of a system. The findings of this study could be used by developers, the government, and stakeholders to capitalize on using the health-related applications with the intention of increasing actual usage. The framework and methodology used presented a way to evaluate health-related technologies. Moreover, the developing trends of using MLA for evaluating human behavior-related studies were further justified in this study. It is suggested that MLA could be utilized to assess factors affecting human behavior and technology used worldwide.

## 1. Introduction

The COVID-19 pandemic has been present for almost three years, starting in March of 2020. Presently, some countries are still suffering from the impact of the pandemic, leaving businesses, industries, and even services closed [1]. One of the countries that is still in strict compliance to help mitigate the spread of the COVID-19 virus is Thailand. Thailand is currently ranked second among the most infected areas of the COVID-19 pandemic in Asia [2].

Thailand was able to develop a mobile application called MorChana to help track the people and critical areas [3]. MorChana is a COVID-19 tracker mobile application that was introduced in early April of 2020. This application has been strictly implemented, and locals and tourists are required to use the application upon entering different areas or vicinities. The mobile application is available both for IoS and Android users [3] and has a push notification setting that informs people when the area has been visited by someone who is positive for the virus. In addition, the application determines risk level, has the ability to let users do self-assessment, as well as to contact people based on the information given. People would also be notified through color-based indications of high-risk level users visiting an area for the past 14 days.

A QR code is given to every user for their information which could be utilized by the health authorities. The use of MorChana has helped the country plan and strategize on developing methods of communication among Thais and healthcare professionals [4]. Viwattanakulvanid [4] added how the reduction of risks, managing and controlling the spread of the virus, and mitigating the rise of cases were prominent upon the utilization of the MorChana [2]. However, this relatively new mobile application was strictly implemented and it was indicated by Reinecke and Bernstein [5] that this does not necessarily have a positive effect among users. Therefore, similar to other related COVID-19 contact tracing applications such as StaySafe from the Philippines, Alipay in China, Tetamman, Tabaud, and Tawakkalna in Saudi Arabia [6] and Aarougya Setu in India [7], these mobile applications should be evaluated and explored to continuously promote their effectiveness with the help of people’s intention to utilize this health-related technology [8]. Since this is a relatively new technology, no studies were seen that evaluated its usability leading to actual usage. In addition, no studies were found relating to MorChana, its technology usage, and people’s behavior in using it.

Theories such as the Unified Theory of Acceptance and Use of Technology (UTAUT2) and the Protection Motivation Theory (PMT) are two of the strong theories that could be utilized when evaluating health-related technology in relation to human behaviors. Several studies have utilized the UTAUT2 for evaluating factors affecting the intention to use a new technology. Venkatesh et al. [9] highlighted how the UTAUT2 framework is one of the complete frameworks to evaluate intention to use a new technology. Their study also suggested to expand the relative factors depending on the purpose of technology. To this aim, this study considered the integration of PMT latent variables to holistically measure the actual usage of a new health-related mobile technology. Ong et al. [10] suggested that PMT is a framework used to measure the coping and threat appraisal of health-related behaviors. The exploration to integrate PMT with another framework has been suggested to holistically measure health-related behavior and the integration of another theory would completely measure human behavior [10].

Several studies have considered utilizing UTAUT2 as a framework to evaluate health-related applications. Alam et al. [11] utilized UTAUT2 to measure mobile health (mhealth) applications. They suggested considering extending the UTAUT2 by incorporating trust, hedonic motivation, self-efficacy, and privacy. To this end, their study presented trust and hedonic motivation as key significant factors affecting intention to use mhealth applications [11]. Consequently, Badran [12] utilized the UTAUT2 of ehealth applications. Their study covering ehealth in Egypt showed how effort and performance expectancy, facilitating conditions, and price had a significant effect on the intention to use the new technology. In addition, Mamra et al. [13] considered the integration of UTAUT2 and PMT to evaluate the acceptance of user-health records from Malaysia. Mamra et al. [13] justified that integrating both theories could holistically measure human behavior covering health-related applications. In this vein, PMT and UTAUT2 integration was also considered in this study.

PMT is a framework that aims to measure the coping appraisal and threat appraisal of health-related studies [14]. Prasetyo et al. [14] considered the integration of PMT and TPB in measuring the effectiveness of community quarantine in the Philippines. Their study highlighted that understanding the risks and vulnerability presented key factors in how people would perceive the protocols as effective. In addition, Ong et al. [15] integrated similar theories to measure the acceptance of a technology. Similarly, their study showed that if people would understand the benefits the technology may present, they would have high levels of acceptance. Moreover, several studies have shown that people would trust a technology when there is less threat and high privacy upon using it [16,17]. Consequently, Yu et al. [18] integrated PMT and UTAUT2 in measuring factors affecting the intention of using mhealth education. Therefore, the use of the integrated theories is justified to completely measure the intention of utilizing the health-related applications, which will lead to an effect on actual usage.

This study aimed to measure the actual use of the COVID-19 contact tracing mobile application, MorChana in Thailand. Several factors such as Performance Expectancy, Effort Expectancy, Social Influence, Facilitating Conditions, Hedonic Motivation, Habit, Perceived Risk, Self-Efficacy, Privacy, Trust, Understanding of COVID-19, Intention to Use, and Actual Use were evaluated in this study. This study considered utilizing machine learning algorithms (MLA), specifically Random Forest Classifier (RFC) and Artificial Neural Network (ANN). Recently, several studies have considered evaluating human factors and human behavior using structural equation modeling (SEM). However, Fan et al. [19] criticized the use of SEM due to the limitations when indirect effects are considered in the model. If the latent variable considers a mediating effect, there may be an effect on the coefficient which represents significance (i.e., lower or insignificance) [19,20]. Woody [20] also highlighted how the mediating variable causes a high level of degradation on coefficient weights, leading to low importance of a latent variable which may be, in reality, higher. Thus, the employment of MLA in studies has been evident.

Taking the study of Alam et al. [11], for example, their study considered the integration of SEM and ANN to evaluate factors affecting intention to use the mhealth application. Their study justified that ANN could promote the identification of different significant latent variables. In addition, Chen et al. [21] highlighted the use of RFC to classify factors affecting human behavior. Their study presented a high level of accuracy in determining factors affecting human behavior. Similarly, Milani et al. [22] considered utilizing decision trees to classify factors affecting human behavior. Moreover, Al-Mashraie [23] showed how using MLA has high predictive powers on behavioral analysis when evaluating technology. Thus, employing MLA could be effective and justifies the limitation of SEM when classifying and predicting factors affecting human behavior such as intention to use and the actual usage of technology. This study is considered the first study that evaluated factors affecting the actual use of the MorChana COVID-19 contact tracing mobile application. The methodology and results of this study could be applied and extended to measure other health-related mobile applications. The framework of this study could also be utilized to measure the usability of different technology and the acceptance of it in other countries. Lastly, the MLA employed in this study could be utilized to evaluate other human-related behavioral studies worldwide.

Following the study of Jamshidi et al. [24], they thoroughly evaluated several studies involving a hybrid of different techniques. Their study covered a wide range of methodologies, techniques, and applications with regard to works of literature applied and considered COVID-19-related issues. Specifically, this included the use of MLAs and Deep Learning. Their study deduced the complex phenomena such as nonlinear, complex, and uncertainty, to which hybrid tools could evaluate efficiently through accuracy of prediction and are reliable. However, Jamshidi et al. [24] highlighted that the further need for validation should be done since this new type of analysis is not yet ideal. To narrow down the scope and application, presented in Table 1 are the different studies with regards to COVID-19 contact tracing applications, country of origin, methodology utilized, purpose, and findings.

It was evident from the presented reviews that the generalizability of the COVID-19 contact tracing is difficult to consider due to several factors involving language, location, understanding, utility, implementation, and complexity, which are factors affecting the perceived usability. As suggested, the need to heighten the individual’s motivation to use the technology will increase adoption and usage rates. The need to assess the COVID-19 contact tracing mobile application individually among users is therefore needed. This will help developers, users, and the government to enhance the motivation for usage, which will lead to mitigating the spread of the COVID-19 virus.

## 2. Related Studies and Theoretical Framework

### 2.1. Application Usability and Related Studies

Several studies have evaluated health-related technologies and their usability. Taking, for example the study of Chuenyindee et al. [8], their study evaluated the contact tracing application, Thai Chana. Their study highlighted that contact tracing that is strictly implemented for usage would lead to users’ dissatisfaction and implementers would see results such as a lack of effectiveness. In line with this study, MorChana is a COVID-19 contact tracing mobile application that has been strictly implemented to use in regions of Thailand. From the study [8], they evaluated the utility of perceived usability among users and presented that perceived usefulness had the highest direct effect on attitude towards using the application which would lead to perceived usability. However, their study highlighted how understanding would lead to the indirect effect of perceived usability. To which, an extension of the study utilizing MLA ensembles such as neural network and RFC was conducted to highlight the most influential factor. The results of the extended study were seen to highlight the perception of severity and how perceived ease of use affected the perceived usability of the mobile application [42]. The study posited that using an MLA ensemble would be beneficial in directly identifying factors affecting perceived usability and actual use due to the limitations set by multivariate tools such as SEM.

Albastaki [37] assessed the perceived usability of contact tracing for COVID-19 in Bahrain. However, they focused on using human task performance measures, a technology acceptance model, and a system usability scale. They highlighted the usability in general rather than factors affecting behavior for utility. On the other hand, Storni et al. [38] evaluated frameworks used to assess the usability of contact tracing applications. They highlighted that usability pillars such as satisfaction, availability, accessibility, flexibility, effectiveness, interaction, and ongoing application evaluations were factors that needed to be considered. Winter et al. [39] considered eye-tracking and a retrospective think-aloud approach for evaluating the German contact tracing application. With the study conducted, they have only concluded that the application is promising and privacy policy is the most significant factor affecting its usability. Blacklow et al. [40] analyzed the COVID-19 contact tracing application in the United States. However, they focused on thematic analysis to evaluate the usability which lacked a lot of analysis to be generalized among other contact tracing mobile applications.

Bente et al. [41] considered the CoronaMedler application and used the think-aloud usability test and eye-tracking. The Netherlands COVID-19 contact tracing was seen to be easy to use, but several demographics found it difficult to interpret, had low trust in privacy, and has been evaluated as underprepared. The need to assess the different usability of these applications should therefore be explored since several studies with different methodologies have been conducted, however, they presented ungeneralizable results. With different COVID-19 contact tracing available and different methods in different countries, the evaluation may be deemed special per type since it is affected by language, culture, and perception of the general users. Yuduang et al. [10] considered the MorChana COVID-19 contact tracing mobile application. However, the analysis using SEM was seen to evidently affect the results due to the presence of a full mediating effect. The highest significant factor was seen to affect actual usage was the intention to use. Other factors were deemed insignificant such as perceived risk. This may come from the presence of mediators are supported by studies that discussed the limitations of SEM [19,20]. They also highlighted that SEM was a limitation and suggested using a machine learning ensemble to validate the findings of the study [10]. Machine learning algorithms are not being utilized to evaluate human behavior and human factors [43].

### 2.2. Machine Learning Algorithm and Related Studies

The abundant data available at present is considered to be a vital source of information. Machine learning algorithms are of different types, wherein classification tools are commonly utilized for pattern recognition. It was stated from the study by Ong et al. [42,43] how MLAs such as RFC and ANN have been recognized in the field of human factors to evaluate human behavior. However, Jamshidi et al. [44] highlighted that the use of artificial intelligence and machine learning can be debatable when it comes to their application since their use is highly dependent on human input, the role of the individual, and collaboration in the roles of human. To this they indicated that data scientists are the ones responsible for the code and that its highlight, limitations, and capabilities should be left to those who code the algorithm. Despite this, several studies have challenged the utility of machine learning as an efficient tool either as a hybrid with other statistical and multivariate tools, or as a standalone measurement and predictive tool. Taking for example the study of Milani et al. [22] and Chen et al. [21], they considered that decision tree and RFC to classify several factors affecting human behavior such as the decision on the removal of children due to familial risk factors and risk evaluation among areas prone to natural disasters. Since MLAs were developed in accordance with statistical tools, it was seen that the consideration of MLA as an alternative tool to replace traditional statistical analysis has been the trend [44]. With the presented studies [21,22,23,42,43], it could be deduced that higher accuracy for the classification model would be obtained for RFC as compared to the basic decision tree. In addition, other classification tools are also available such as different types of regression. However, these tools were basically created before the more advanced algorithms.

Other tools such as neural networks have also been considered. Daube et al. [45] expounded on how a neural network resonates with the receptor the human body has. These neural connections can then be connected to calculate and recognize patterns which eventually leads to classifying affecting factors. Jamshidi et al. [46] considered artificial intelligence such as deep learning to evaluate and accelerate the COVID-19 vaccine design, considering also the application of which other vaccines utilized worldwide. Their study proposed how neural networks are highly complex algorithms that could be applied in health-related studies. Yariyan et al. [47] utilized ANN to evaluate risk assessment among citizens in Iran. Oktarina et al. [48] considered ANN to determine casualties and damages among people in Indonesia. It could be deduced from these studies how ANN is considered a cutting-edge algorithm that can calculate complex connections, producing an accurate result of prediction [49]. Other classification models could be compared to it, such as K-Nearest Neighbor and Naïve Bayes. However, Ong et al. [42] explained how these classification models are more complex and would be applicable for more complex frameworks. Lastly, clustering algorithms such as K-Means may also be considered, that is if the aim of the study is to create groupings of indicators or items affecting a dependent variable.

With that being said, the goal of this study aims to predict factors affecting actual usage of the MorChana mobile application, and it was posited to consider the ANN and the RFC ensemble. Similarly, Ong et al. [43] considered the same ensemble to predict factors affecting the acceptance of a technology by a population. It was seen that these algorithms would be best to predict factors creating a higher accuracy rate with less complex algorithm and calculation. Jamshidi et al. [44] indicated that similar proposed techniques have been underexplored with regard to its application; however, its efficiency and accuracy are relatively reported to be effective. In another study, Jamshidi et al. [24] concluded that these methods play vital roles in predicting and forecasting outputs in relation to their target in terms of complex behavior of phenomena functions. In addition, it was stated by Kheirollahpour et al. [50] explained how pattern recognition from the nonlinear relationships may be classified by neural networks. The presence of hidden layers would help calculate the nonlinear relationship present and thus can identify the relationship present. As support, Ong et al. [42] also considered the neural network and RFC ensemble to predict factors influencing the perceived usability of a mobile application. It was deduced that a deep learning neural network may contribute to a higher accuracy rate if the model considered is quite complex. Therefore, an ensemble of ANN and RFC was utilized in this study following several studies [21,22,23,42,43,44,45,46,47,48,49,50].

### 2.3. Theoretical Framework

**Figure 1:**
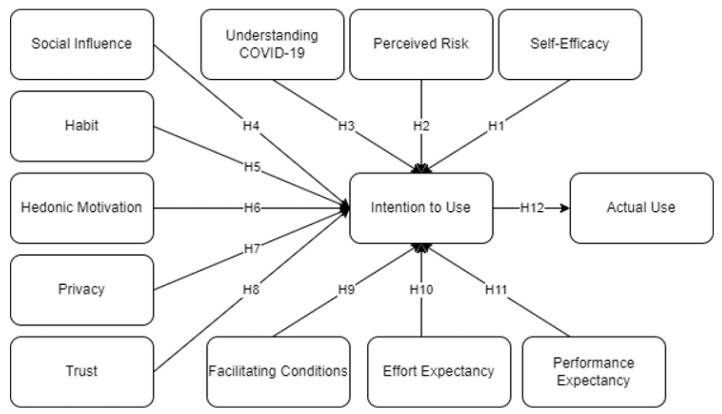
Theoretical Framework.

The theoretical framework of this study is presented in Figure 1. Under PMT, several factors such as understanding COVID-19 (U), Perceived Risk (PCR), and Self-Efficacy (SEF) were considered as extended factors for UTAUT2. These factors were considered as representations of threat and coping appraisal behaviors, respectively [10,14,15]. Under UTAUT2, several factors such as Social Influence (SI), Habit (HB), Hedonic Motivation (HM), Privacy (PR), and Trust (TR) were considered alongside the PMT variables to measure intention to use (IU) the MorChana COVID-19 contact tracing mobile application leading to Actual Use (AU) of the mobile application. Following several studies that suggested and integrated both PMT and UTAUT2 [9,11,13], this study aimed to evaluate which factor significantly affects AU of a health-related mobile application, MorChana.

PMT factors such as U, PCR, and PT were seen to have a significant effect on IU. Ong et al. [10] showed how U presented to be one of the most significant factors affecting a positive behavior towards mitigation when threat among people is evident. Their study presented that when health and well-being are considered, people would highly consider their safety due to their understanding of the event. In addition, another study [15] showed how the acceptance of technology would be based on people’s understanding. The benefits of a technology will outweigh threats if people understand how it could positively affect their lives. Moreover, if PCR is evident, people would have the intention to mitigate all possible appraisals [14]. Featherman and Pavlou [51] explored the risks evident when adopting services. Their study found that eservices are affected greatly by the PCR, and reducing the risks would lead to higher IU. In addition, Thakur [52] justified that SEF will greatly affect satisfaction, leading to a high and positive effect of a mobile application on IU. Several studies have also justified how these factors greatly affect IU [13,53]. Therefore, the following were hypothesized:

**Hypothesis** **1** **(H1).** *U has the most significant effect on IU leading to AU.*

**Hypothesis** **2** **(H2).** *PCR has the most significant effect on IU leading to AU.*

**Hypothesis** **3** **(H3).** *SEF has the most significant effect on IU leading to AU.*

SI is the influence of other people on a person using a technology [9]. Venkatesh et al. [9] presented how the early stages of a developing mobile technology would consider SI as one of the key factors to having high IU. Alam et al. [11] explained how people without prior knowledge would have difficulty using the application. The utility of a mobile application would have high IU if people around the user have experience in using the application, leading to the influence due to the confidence of available assistance and available knowledge. Ezzaouia and Bulchand [54] also showed how SI is one of the significant factors affecting the IU of a COVID-19 contact tracing application upon utilizing the UTAUT2 framework. Thus, it was hypothesized that:

**Hypothesis** **4** **(H4).** *SI has the most significant effect on IU leading to AU.*

HM and HB were indicated to be significant factors affecting IU of technology [55]. Nikolopoulou et al. [55] explored e-education, focusing on the acceptance of technology. When people constantly use a certain technology, HB is developed [56]. The study of Chen et al. [56] explained how the consistent usage of technology would lead to a loop, especially when dealing with health-beneficial applications. Thus, a high IU would be seen as preceded by HB and if users find it fun, enjoyable, and easy to use, then it would lead to high IU as well [56]. In addition, Amoroso et al. [57] indicated that when HB precedes satisfaction among users, this leads to a positive IU. It was therefore hypothesized that:

**Hypothesis** **5** **(H5).** *HM has the most significant effect on IU leading to AU.*

**Hypothesis** **6** **(H6).** *HB has the most significant effect on IU leading to AU.*

Upon the utility of technology, people would consider the PR and TR when considering which technology to use [58]. It was indicated in the study of Martins et al. [59] that IU is preceded by the user’s control over their personal information and protection. Both the lowered risk and higher trust in a system would lead to a positive IU [58,59]. In addition, Mingxing et al. [60] justified that when PR is not evident, and TR has been built on among users, continuous utility and engagement of AU would be evident. Moreover, Lallmahomed et al. [61] showed how TR would have a positive effect on IU, and higher PR would lead to a negative IU among users. Thus, it was hypothesized that:

**Hypothesis** **7** **(H7).** *PR has the most significant effect on IU leading to AU.*

**Hypothesis** **8** **(H8).** *TR has the most significant effect on IU leading to AU.*

Under UTAUT2, FC, EE, and PE latent variables were seen to have a key significance when evaluating and using a technology [9]. Due to the continuous rise and effects of the COVID-19 pandemic, mobile applications have been utilized and has been considered by people to help in reducing the risk of exposure [8]. These factors were also seen to be positively affecting the IU, leading to AU in Belgium [62]. Walvare et al. [62] showed how PE followed by EE were significant factors for users to use a particular technology. They highlighted how these factors can significantly affect IU under UTAUT2, but generalized it to technology users. In addition, Alam et al. [11] explained how more utility of the mobile application would lead to higher IU. They expounded on the mobile adoption of users and that UTAUT2 factors such as PE, EE, and FC affect the IU significantly, leading to AU. Thus, it was hypothesized that:

**Hypothesis** **9** **(H9).** *FC has the most significant effect on IU leading to AU.*

**Hypothesis** **10** **(H10).** *EE has the most significant effect on IU leading to AU.*

**Hypothesis** **11** **(H11).** *PE has the most significant effect on IU leading to AU.*

IU is the measurement of performing a particular action [10]. Wu and Du [63] explored the behavioral intention and system usage constructs. Their study showed that there is a positive correlation on IU and AU. When dealing with health-related applications, goods, and technologies, Dehghani [64] presented how AU is preceded by IU. This is supported by the study of Huang and Yang [65], wherein they suggested that IU and AU have a significant relationship when dealing with health-related technology. Therefore, it was hypothesized that:

**Hypothesis** **12** **(H12).** *IU has the most significant effect on AU.*

## 3. Methodology

### 3.1. Questionnaire

The table of constructs is presented in our previous study [10]. From the constructs, several indicators were considered to evaluate factors affecting the actual usage of the MorChana COVID-19 contact tracing mobile application [10]. Several factors under the UTAUT2 were considered, such as performance expectancy, effort expectancy, habit, hedonic motivation, social influence, facilitating conditions, privacy, and trust. Moreover, latent variables from the PMT were considered such as understanding the perceived risk of COVID-19 and self-efficacy. Adapted from several studies, there were a total of 53 indicators considered in this study.

Utilizing a 5-point Likert scale to evaluate the different factors, a preliminary test of the questionnaire was conducted. With 150 responses collected, the Cronbach’s alpha resulted in a value of 0.856. Following the suggestion of Hair [66], the questionnaire is deemed valid and could be considered for distribution.

### 3.2. Participants

This study was approved by Navaminda Kasatriyadhiraj Royal Thai Air Force Academy’s Research Ethics committees and the School of Industrial Engineering and Engineering Management of Mapua University’s Research Ethics committees. Utilizing an online survey for data collection, convenience sampling was employed to gather 907 valid responses from volunteer participants. The 907 participants were able to utilize the MorChana mobile application to ensure that the response obtained may be considered for this study. Prior to the analysis of the datasets considered, SPSS 25 was utilized to determine any missing response. No missing data was seen. In addition, the Harman’s Single Factor test for common method bias was conducted for the analysis of bias and false responses. It was stated from the study by Podsakoff et al. [67] and Hair [66] that a threshold of 50% would result in no bias and false responses. This study was able to obtain a result of 36.42%, which is below the set threshold. Thus, the data collected is acceptable with no missing data, false responses, and bias. A total of 48,071 datasets were considered for the MLA analysis.

### 3.3. Machine Learning Algorithm

**Figure 2:**
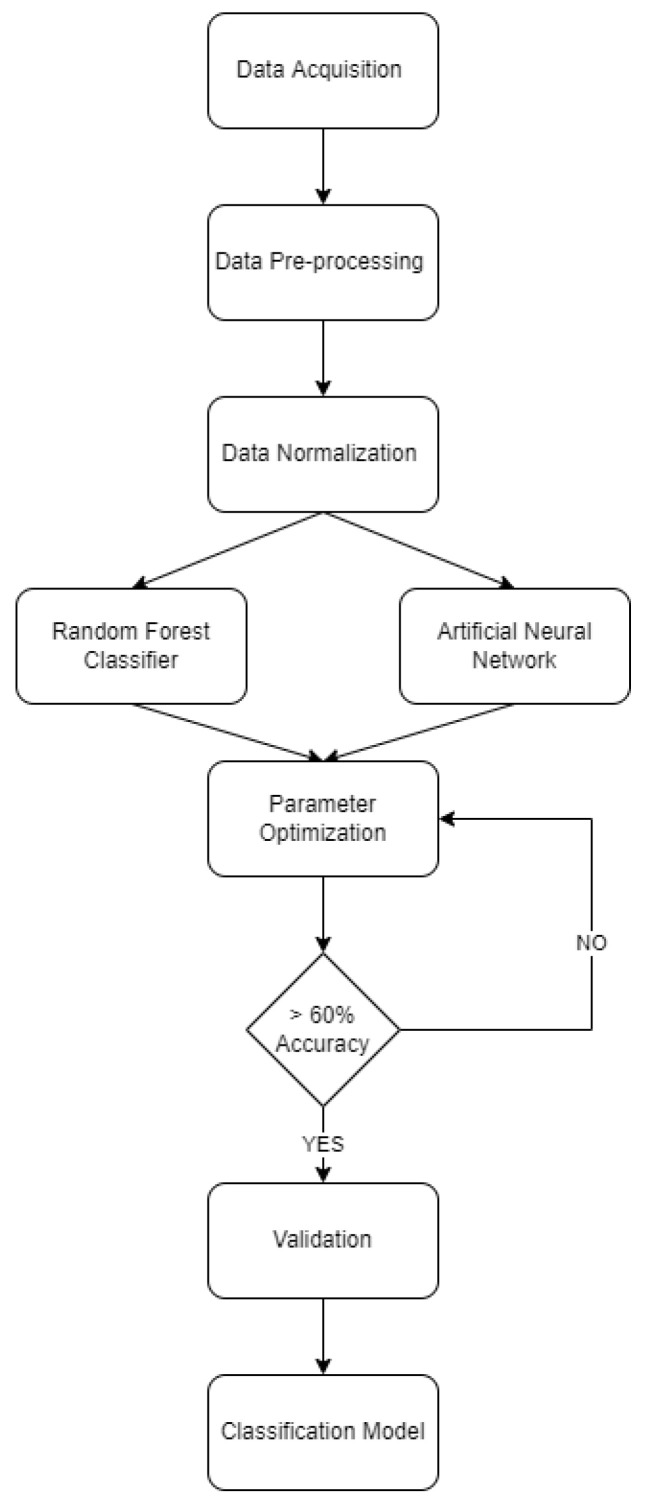
Methodological Flowchart.

Presented in Figure 2 is the methodological flowchart utilized in this study. Data acquisition was the first thing done, followed by data pre-processing and data normalization. The dataset was then considered as the input variables for both machine learning algorithms. Parameter setting and optimization was conducted, followed by the validation and generation of a classification model. Lastly, result interpretation was conducted.

Data pre-processing was employed for the 48,071 datasets. Data cleaning using correlation analysis was conducted [42,43]. Indicators with a *p*-value greater than 0.05 and a correlation coefficient less than 0.20 were considered insignificant. Ong et al. [42] indicated how weak correlation will still be considered for the analysis. However, anything lower than the threshold would be deemed insignificant and should not be considered since values lower than 0.20 would be considered as no correlation and are negligible [68]. Based on the results, all indicators were considered significant, and thus data aggregation was conducted. Averaging the indicators to represent each factor was conducted. The aggregated datasets were then normalized to process the RFC and ANN. Chen et al. [21] presented how RFC is a type of MLA that produces a classification model with high accuracy.

#### 3.3.1. Random Forest Classifier

RFC was developed in Python 4.5. The algorithm considered utilized packages such as pandas, sklearn, with its ensembles, and graphviz. The RFC would generate a tree every run as the classification model based on set parameters adapted from the study of Ong et al. [42,43]. Different factors such as HB, HM, FC, U, PE, EE, PCR, PR TR, SI, FC, and IU were considered to analyze the factor affecting actual usage of the MorChana COVID-19 contact tracing mobile application. The different factors were then utilized to run the RFC optimization considering different parameters. Parameters such as the splitter (gini or best), criterion (random or best), tree depth (4–7), and training and testing ratios (60:40–90:10) were considered to determine the optimum tree. Utilizing python 4.5, 100 runs per combination was considered to determine the best tree. A total of 6400 runs were conducted.

The study of Yang and Zhou [69] explained how the different splitters would create a homogeneous dataset considered in the algorithm. In addition, the reduction of impurities would be evident upon the utilization of either gini or entropy [42]. In addition, the criterion of how the leaves would be split would be based on either choosing the best output or randomly. The consideration of tree depth would be necessary for generating the tree to reduce both overfitting and underfitting. Ong et al. [43] discussed how having a lower depth of tree may cause underfitting of tree results while having a larger tree may produce overfitting. Lastly, determining the training and testing ratio would be a significant contribution to produce a consistent tree. This determines the significant factors affecting a dependent variable. The RFC pseudocode is presented as Algorithm 1:
**Algorithm 1:** Random Forest Classifier*Step 1.* Loading of pre-processed dataset. *Step 2.* Setting and Splitting the dataset among training and testing utilizing train_test_split from sklearn.model_selection.*Step 3.* Setting the tree depth and random state at 0. *Step 4.* Setting the criterion and splitter parameters with RandomForestClassifier from sklearn.ensemble. For Gini, Equation (1) was utilized while Entropy considered Equation (2). p = probability of the factor classified to a particular class from j to t.**For gini index:**#The algorithm works at 1 − (probability of the 1st class squared + 2nd class squared + … n)   For each split of branch:       Percent branch representing the used weight will be calculated       For each class:          Calculation of probability in the branch given          Probability will be squared   Take sum of squared probability of class   Subtract of summation from 1 to get gini index for each branchObtain weight of each branch from baseline probabilityTaking sum of all weighted gini index for each split.**For entropy index:**#The algorithm works at [*p* 1st class * log(*p*(class1),2) + *p* 2nd class * log(*p*(class2),2) + … n].   For each split of branch:       Percent branch representing the used weight will be calculated       For each class:          Calculation of probability in the branch given          Probability of class will be multiplied with log (probability, base = 2)          −1 will be multiplied with the result       Taking the sum of the resulting probabilities   Obtain weight of each branch from baseline probability   Taking sum of all weighted gini index for each split.*Step 5.* Setting the splitter parameters (random or best) with RandomForestClassifier from sklearn.ensemble.*Step 6.* Creating the confusion matrix using classification_report, confusion_matrix from sklearn.metrics. *Step 7.* Printing the classification report and decision tree with random forest classifier using graphviz from sklearn.tree. Generation of training and testing accuracy result, precision, recall values, and run time will be obtained.

(1) $$Gini\;\left(t\right)=1-\Sigma_{j}{\left[p\left(j|t\right)\right]}^{2}$$

(2) $$Entropy\left(t\right)=-\Sigma_{j}p\left(j|t\right)\log p(j|t)$$

where: *p* = probability of the factor classified to a particular class from *j* to *t*.

#### 3.3.2. Artificial Neural Network

**Figure 3:**
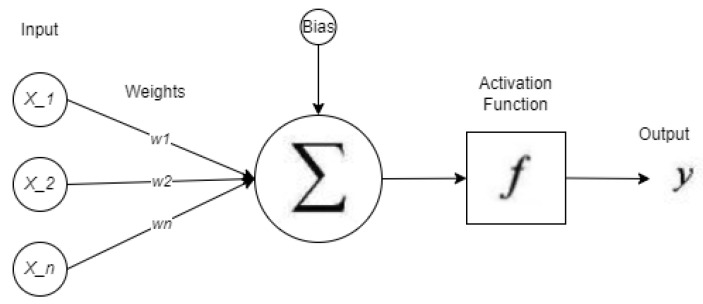
ANN Operation.

ANN data pre-processing was conducted utilizing correlation analysis, similar to the pre-processing technique employed with RFC [42,43]. Optimization of ANN parameters were conducted to determine the optimum model. The activation functions such as swish, relu, and tanh for the hidden layer was considered. For the output layer, sigmoid and softmax were considered. Lastly, optimizers such as adam, RMSProp, and SGD were considered [42,43]. Represented in Figure 3 are the operations done by the ANN.

Determining the optimum hidden layer nodes, each run considered increments of 10 nodes until 100 runs were reached. Adopting the study of Pradhand and Lee [70] and Walczak and Cerpa [71], 10 runs of each combination were conducted using 150 epochs. A total of 9900 runs were considered for the ANN optimization. The ANN employed feed-forward processing using an 80:20 training and testing ratio. Packages utilized for ANN were SKLEARN with numpy, pandas, matplotlib.pyplot, tensorflow and keras, and train_test_split. The pseudocode for ANN is presented as follows Algorithm 2:
**Algorithm 2:** Artificial Neural Network*Step 1.* Loading of preprocessed data.*Step 2.* Feature selection was set for dependent and independent variables. *Step 3.* Setting and Splitting the dataset among training and testing utilizing train_test_split from sklearn.model_selection with 0 random state.*Step 4.* Utilizing keras sequential for the number of nodes and parameters for the input layer, hidden layer, and output layer.#For Activation Function   #Hidden Layer swish equation       Swish (Y) = X * Simgoid (X)        Swish is considered a more efficient type of activation function following the popular ReLu since it is a continuous smooth function. This type of activation function follows negative weights with small values for its propagation process. Usually, swish is utilized for a successful nonmonotonic type of activation function. Its trainable parameter offers fine tuning for maximized output of propagation with a smooth gradient. This generates a faster, easier, and efficient generalizability of results [72].   #Hidden Layer ReLu equation       Rectifier Linear (ReLu) = (f(x) = max (0, x))       ReLu thresholds all negative values to zeroes and has consistent gains compared to other activation functions. It has been deduced that ReLu is established as an activation function that is consistent even with deep learning neural networks. Especially with nonlinear models, ReLu is an activation functions that can surpass the nonlinearity model and can calculate the relationship with high accuracy. In addition, ReLu has been seen to have low computation complexity when utilized and is versatile [73]. This was also considered and utilized in other studies [74,75,76].    #Hidden Layer Tanh equation$\;\tanh\left(x\right)=\frac{2}{1+e^{-2x}}-1$       Tanh is considered to be a shifted and stretched sigmoid function which performs better since it considers the negative values as compared to the automated zeroes from sigmoid. Gudivada and Rao [77] explained how neural networks consider activation functions such as Tanh to calculate nonlinear relationships provided that the hidden layer and nodes are optimized, suitable to the model considered. Tanh have greater gradients resulting to stronger calculation of updating the weights. With that, a faster optimized result will be obtained. A nonlinear relationship may be easily resolved and identified with Tanh [62]. Several studies [78,79] have utilized Tanh for its effectivity.   #Output Layer Sigmoid equation$\;Sigmoid\;\left(x\right)=\frac{1}{1+e^{-x}}$       The sigmoid activation function is used for its capabilities to calculate nonlinear and bounded values with a smaller range. Negativity is set and calculated as zeroes. For output layers, processing of data has been accomplished at the hidden layer, and thus are forwarded to the nodes as smaller values [42]. Sigmoid functions are usually utilized to its simple but efficient calculation. It is commonly used in the output layer since its results are interpreted as probabilities [80,81].   #Output Layer Softmax equation$\;softmax\;\left(z_{j}\right)=\frac{e^{z}j}{\sum_{k=1}^{K}e^{z}k}$       Softmax is similar to sigmoid but the denominator is summed. The advantage of using softmax is it takes into account all output compared to the individual one like sigmoid. *Step 5.* Setting parameter for the optimizer [42,43]. Unlike sigmoid, the softmax results are all interrelated and by design, the result of softmax will be the probability with a sum equal to 1. The likelihood of one class will be decreased if one class needs to be increased. This is ideal for a nonlinear relationship and output layers since it uses a multi-classification calculation. In addition, softmax may also be considered for the activation function in the hidden layer, which shows its versatility [82]. *Step 6.* Setting parameters for optimizer and number of epochs.*Step 7.* Feedforward process (learning rate, bias, weight (w)) considers the equation,$\;Y=\overset{m}{\underset{i=1}{\sum }}\left(x_{i}*w_{i}\right)+b$   Where:$\;x_{i}$ = input features$\;w_{i}$ = weights   *b* = bias    Then the activation function (*f(Y)*) is applied for the output, output=f(Y).   The calculation pseudocode is as follows:   OutputB = 1st input*w [0] + 2nd input*w [1] + bias*w [2]   If OutputB > 0: #Activation Function considered       OutputB = 1   Else       OutputB = 0   Error = output–OutputB   W [0] += error * 1st input * learning rate   W [1] += error * 2nd input * learning rate   W [2] += error * bias * learning rate   OutputB = #calculation using the activation function *Step 8.* Printing of validation test results. Generation of training and testing accuracy result, precision, recall values, loss rate, and run time will be obtained.

## 4. Results

### 4.1. Participants

**Figure 4:**
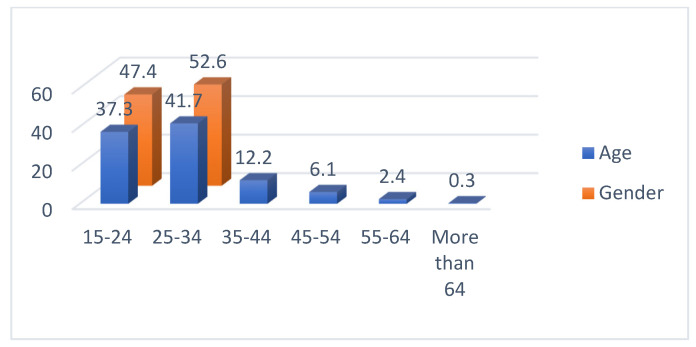
Descriptive Statistics of Age and Gender.

**Figure 5:**
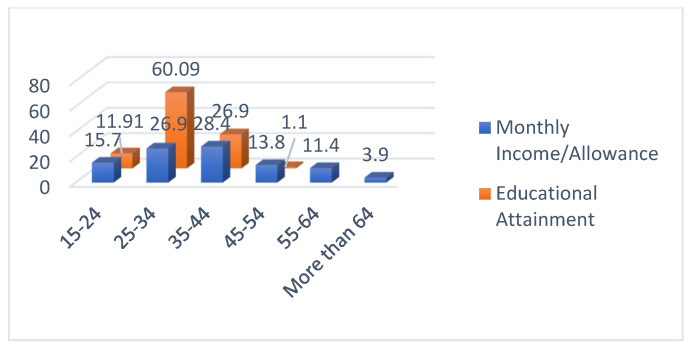
Descriptive Statistics of Income and Education.

The descriptive statistics of the respondents are presented in Table 1. From the results, 47.4% were female and 52.6% were male, ranging from 15–24 years old (37.3%), followed by 25–34 years old (41.7%), 35–44 years old (12.2%), and the rest were older, as represented in Figure 4. The majority of the respondents have a bachelor’s degree (60.09), followed by a master’s degree (26.90%), then a high school degree (11.91%). The majority earns either 10,000–20,000 THB (26.9%) or 20,000–30,000 THB (28.4%), followed by less than 10,000 THB (15.7%), and the rest are greater than 30,000 THB, as represented in Figure 5. Lastly, the majority are not covered by COVID-19 insurance (89.4%).

In addition, the validity of the constructs through Cronbach’s alpha is presented in Table 2 together with the VIF values for the multicollinearity test. Following the suggestion of Chuenyindee et al. [8], no multicollinearity is seen since the VIF values are less than 5.00. Thus, the constructs utilized in this study are considered to be valid without multicollinearity.

### 4.2. Machine Learning Algorithm

Presented in Table 3 is the result for the RFC that produced the highest accuracy, depth 5. It presents the results from the training and testing ratio from 60:40 until 90:10. It could be seen that the highest result presented a 93% accuracy with a 0.00 standard deviation. In addition, the average accuracy and recall values were also considered. It was seen that at this combination, the highest precision and recall were also obtained.

**Figure 6:**
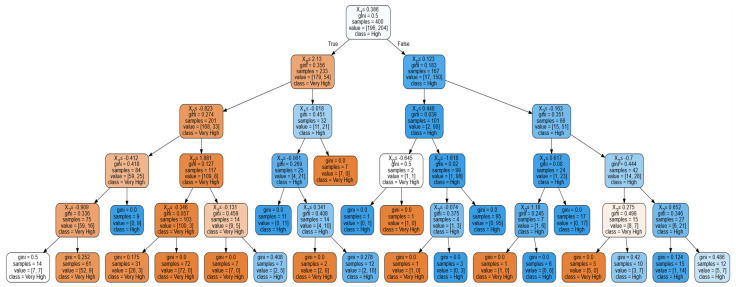
Optimum Random Forest Classifier.

Presented in Figure 6 is the best tree derived from the optimization run. An accuracy rate of 93% with a 0.00 standard deviation was seen upon running the test. The run time per combination took 0.146 s. This indicates that the tree models the classification of significant factors affecting the actual usage of the MorChana COVID-19 tracking mobile application. The parameters that resulted in the best tree were gini for the splitter, best for the criterion, and 5 as the tree depth, using the 80:20 training and testing ratio. The results of the RFC indicate a ‘very high’, corresponding to the 5th point of the Likert Scale utilized, ‘high’ for the corresponding fourth point, and so on.

Based on the tree, it was seen that HM (X1) represents the parent node, considering values less than or equal to 0.386. It will then consider HB (X0). If the value to consider is less than or equal to 2.13, it will consider X0 with values less than or equal to −0.823. Satisfying this will consider X1 and then FC (X2), which will lead to a very high AU. On the other hand, if HB (X0) is not satisfied, it will consider X0, which will lead to a very high AU. If the child node X0 is not satisfied, it will consider X1, then X2, leading again to a very high AU.

On the other hand, if X0 of the child node will be considered, values less than or equal to 0.123 will determine the branch. Satisfying this will consider X1, then X0, which will lead to a very high AU. If 0.123 was not satisfied, it will consider X2, then U (X3), then X2, which will lead to a high AU. Thus, it could be deduced that factors HM and FC would lead to significant and very high AU. HB and U would be significant and would have high AU among MorChana COVID-19 contact tracing mobile application users. However, only significant factors and their effects are evident in the RFC. No level of importance and lower significant factors could be seen from the results. To verify the findings of the RFC, neural networks may be employed. Following the suggestion of Abiodun et al. [83], ANN may be utilized to help determine the importance of significant variables affecting cognitive computing.

**Figure 7:**
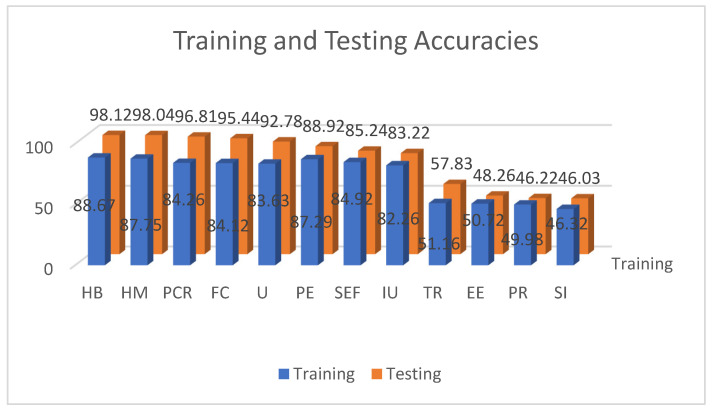
ANN Results of Training and Testing Accuracies.

Figure 7 represents the summary of ANN results from the optimization run. A 60% threshold was set to determine the significant level of the results from testing ratios [42,43,71]. From the results, HB had the highest average testing result, followed by HM, PCR, FC, U, PE, SEF, and IU. These factors were ranked based on the average testing results, representing the most to least significant.

**Figure 8:**
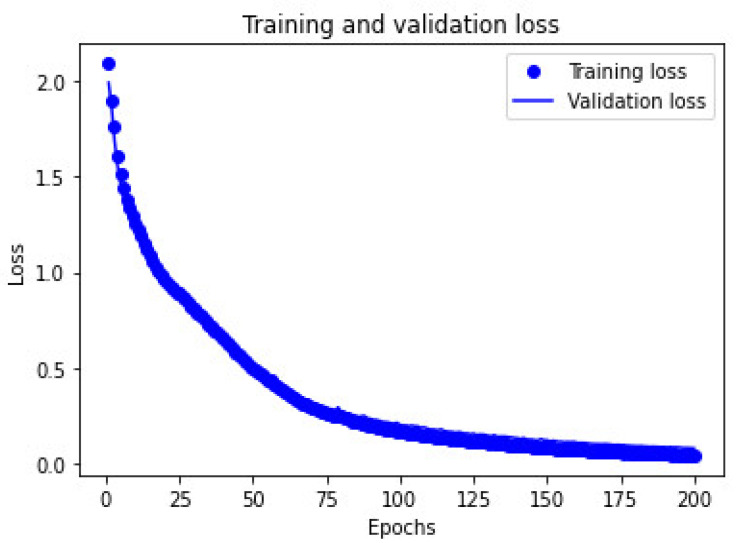
Training and Validation Loss of ANN.

An analysis of variance was conducted among the results to determine the significant differences from the initial run. The highest value was considered for the final parameters of creating the ANN. Ten nodes for the hidden layer with relu as the activation function, together with the softmax for the output layer activation function, and adam as the optimizer was utilized. Using 200 epochs at 70:0 and 80:20 training and testing ratio, 50 runs were conducted and the average result presented a 98.15% result with 1.193 standard deviations at 80:20. Recall and precision values resulted in 97.82 and 98.00, respectively. The run time for each combination for ANN took 0.480 s. The training and validation loss of the ANN run that resulted in no overfitting is presented in Figure 8. Following the study of Walczak and Cerpa [71], this result was found to be relatively high for studies concerning human behavior. Thus, the results are acceptable.

The final ANN includes the different factors as the input nodes with 10 hidden layer nodes, and one node for the output layer representing the actual usage of the MorChana COVID-19 contact tracing mobile application. From this, relu as the activation function for the hidden layer, softmax for the output layer, and adam as the optimizer would lead to an accuracy rate of 98.15%.

**Figure 9:**
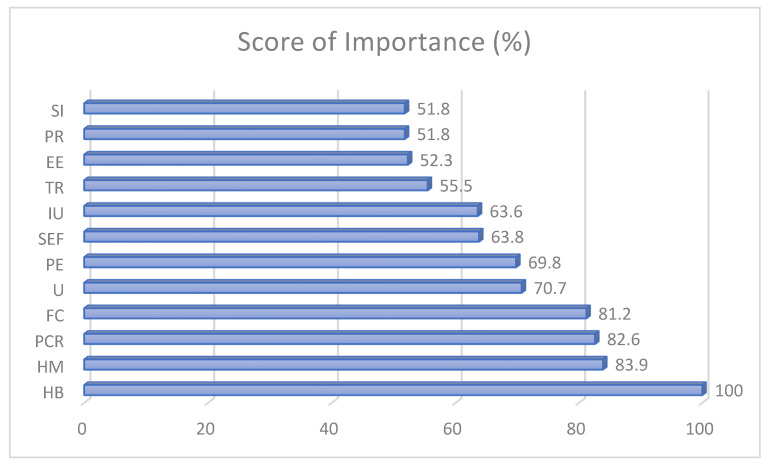
Score of Importance.

To validate the MLA results, the score of the importance and correlation analysis was conducted in SPSS 25. Presented in Figure 9 is the score of the importance of the different factors. Similar to the results of ANN and RFC, HB presented as the highest significant contributing factor for actual usage of the MorChana COVID-19 contact tracing mobile application. The second highest was HM, followed by PCR, FC, U, PE, SEF, and IU. From the results, TR, EE, PR, and SI were the least important factors affecting the AU of MorChana (score < 60%).

In addition, the correlation analysis results are presented in Table 4. All values presented in the table have *p*-values less than 0.05, and thus are significant. From the results seen in the AU factor, similar rankings are evidently based on the correlation coefficient. This further justifies the findings of the RFC and ANN.

## 5. Discussion

### 5.1. General Findings for Usability

This study considered utilizing RFC and ANN in predicting factors affecting actual usage of the MorChana COVID-19 contact tracing application. With the integration of UTAUT2 and PMT for evaluation of actual usage (AU), factors such as hedonic motivation (HM), Habit (HB), Perceived Risk (PCR), Understanding of COVID-19 (U), Performance Expectancy (PE), Self-Efficacy (SEF), Intention to Use (IU), Trust (TR), Effort Expectancy (EE), Privacy (PR), and Social Influence (SI) were evaluated. As seen from the results, the RFC model generated a 93.00% accuracy with a 0.00 standard deviation. Moreover, ANN resulted in a 98.12% average accuracy with a 1.172 standard deviation. From both results, it was evident that HB, HM, PCR, FC, and U greatly affected the actual usage of MorChana in a positive way. In addition, a Pearson correlation and score of importance validated the findings.

HB was indicated to greatly contribute towards very high actual usage. People in Thailand were seen to have adopted the consistent usage of MorChana, and it has become a regular activity for them. In line with the results of Wu et al. [84], their study presented how the increase in HB also increases the satisfaction level in utilizing an application. In Thailand, people have been consistently using MorChana since early April 2020. With the strict implementation and utility, people have adapted well to its regular use upon entering a certain area. Palau-Saumell et al. [85] explained how continuous usage will eventually develop the habit due to consistent usage. Thus, leading to the highest significant factor affecting AU. In addition, Nikolopoulou et al. [55] showed how both HB and HM are key indicators contributing to an individual’s intention to utilize health-related applications.

HM was seen to be the second-highest factor affecting AU. From the RFC result, this would lead to high AU. The indicators presented how using MorChana led to enjoyment, fun, entertaining, and pleasurable in a health-related context. People would believe that it helps in preventing the COVID-19 spread, thus leading to it being a positive significant factor. Following the study of Palau-Saumell et al. [85], their study presented how the consistent utility leads to people having the pleasure and enjoyment of consistent usage in daily activities. Venkatesh et al. [9] also showed how HM derives the enjoyment in innovative technologies such as COVID-19 contact tracing applications. When people recognize the necessity, a high intention leading to AU would be seen when incorporated into daily activities [9].

Third, PCR was seen to be one of the significant factors affecting the AU of the MorChana COVID-19 contact tracing application. It was seen from the indicators that using MorChana leads to help in assessing symptoms of COVID-19, identifying the area and people at risk of COVID-19, and giving notification when the area is infected. These important factors have been considered as reasons why PCR is an antecedent of AU. The study of Shemesh and Barnoy [86] expounded on how the mhealth application would have a high intention of usage among users when the providers of the application are related to the healthcare system. In addition, Tuman and Moyer [87] explained how mobile health applications would be utilized when benefits to health are seen. Thus, this supports the findings of this study.

Fourth, U was seen to have an important effect leading to very high AU. Based on the indicators, people know how COVID-19 spreads, they know its incubation period, symptoms, and preventions even before the MorChana application was utilized. This indicates that people know how severe and vulnerable they are to the COVID-19 virus. It presents how knowledgeable people are since the COVID-19 virus is prevalent even in other countries [88]. Following the study of Wang et al. [88], they presented how 89.8% of Thais know and understand the effect of COVID-19 when they get infected. In addition, Caldwell et al. [89] explained how the minimum health standard knowledge would reduce and mitigate the transmission of the COVID-19 virus. Therefore, this justifies why having U would lead to a very high AU of the MorChana application since people know the help the technology brings.

Fifth, PE was seen to be a significant and important factor leading to very high AU. Upon using the application, people find it useful, and helps in preventing the contraction of COVID-19. This indicates how there are benefits and advantages to utilizing the MorChana COVID-19 contact tracing application. Venkatesh et al. [9] explained how PE is one of the key indicators for people to accept and have high intentions in using a technology. Consequently, Alam et al. [11] showed how people would have high utility for and would adopt a technology when there is a positive effect caused by PE. Upon dealing with health-related benefits, the higher benefits would lead to a positive effect of PE on AU.

From the ANN results and score of importance, SEF was seen to be an important factor affecting AU. Consequently, IU was also seen to have a positive important effect on AU. People find using the MorChana application to be convenient, easy to use, and having the availability of health services. In addition, indicators of IU present the continuous patronage of the application utility, incorporate the practice of using it daily, and would even install the application when changing mobile phones. Ong et al. [15] indicated how the technical skills and knowledge of people with technology will lead to their acceptance of utilizing it. In addition, Thakur [52] explained how both SE and satisfaction in using an application would have a positive effect on continuous intention in using an application. Chuenyindee et al. [8] also presented a positive significant effect of IU on AU caused by the understanding of people towards the risks of the COVID-19 virus and the benefit of using a COVID-19 contact tracing application. Thus, this supports why SEF and U were evidently important factors affecting the AU of the MorChana COVID-19 contact tracing application.

Interestingly, the ANN presented how factors such as TR, EE, PR, and SI were seen to have low significance and lower scores of importance among other factors. Under TR, people know that the MorChana application is trustworthy, having high reliability, not opportunistic, and that the promises upon using the application are consistent. Subsequently, PR was also seen to have low significance. The indicators showed how the application has protection with personal information, high security, and no trackers placed. As indicated by an article regarding MorChana, the developers were able to promote the usage by highlighting security with information and data collected [3]. From the results, it could be seen that the developers were able to be consistent with their promotion. Thus, people did not give much attention to either factors.

In addition, EE was seen to have a low significant effect on AU. From the indicators, people find the application easy to use, clear and understandable interaction is seen, and they could be proficient with the mobile application. Effort would be lessened if people have already adopted the usage of the technology [15]. Several studies [55,66,69,90] presented how EE is a significant indicator leading to AU of technology. However, the application has been strictly implemented for compliance among people in Thailand. Thus, people easily adopted the technology. To which, it led to a low significant effect and low importance for AU.

Lastly, SI was seen to have the lowest score of importance among all other factors affecting AU. In addition, the ANN presented a very low accuracy result, leading to a low significant effect with AU. From the indicators, people around an individual influence them to use the MorChana mobile application. It makes sense why this is the least significant factor because the government implemented the strict utility of the application, thus no influence by others would be evident. According to Reinecke and Bernstein [5], forcing people to use a certain technology would not automatically lead to a positive AU. However, based on the indicators presented by the significant factors, people find the application easy to use, beneficial, and that it promotes health safety. Therefore, despite the strict guidelines, people would still consider utilizing and promoting the continuous AU. Therefore, this finding should be capitalized on by the government and developers. For people to patronize a technology, the benefit and people’s understanding should be highlighted to have a positive effect on users.

### 5.2. Usability and Willingness among Users

It could be deduced that people developing habits of using the mobile application would lead to an increase in actual usage. With the constant usage and implementation of utilizing the mobile application, people from Thailand were seen to have adopted and grown accustomed to using the application. This has led to the hedonic motivation to use the mobile application. Similar to the study of other COVID-19 contact tracing applications, Tomczyk et al. [91] explained that the behavior of people would greatly affect their usage of these contact tracing mobile applications. It was seen that people’s control of their behavior would influence their actual usage. It could also be posited that strategies should be created to highlight the usage of these contact tracing applications [91]. The findings of this study also indicated that perceived risk and understanding of COVID-19 influenced people’s actual usage. This indicates how people in Thailand understand and have knowledge of the effect of the virus, which led to the usage of the contact tracing application. It was explained [15] that when people consider the benefit and are at high risk (i.e., health-related), an acceptance of technology will be evident. Lastly, PE is a key factor for usability as indicated by the results. Similar to the study of Ezzaouia and Bulchand-Gidumal [54], these factors are significant in the intention and adoption of users for contact tracing applications.

With the challenge faced by the government to encourage people to use the application, it could be advised to have a user-friendly mobile application, and to promote the application for its health benefits and show how it could reduce the risk of contracting the virus. These general findings may then be adopted and applied to other contact tracing mobile applications to encourage usage among citizens. Overall, people find the utility of the MorChana COVID-19 contact tracing application to be a pleasant experience, as it helps in protecting their health and can be used anytime and anywhere. With continuous usage, people will adopt the technology and using it would become a habit. Moreover, the ease of use and the understanding of the health-related benefit would create a very high AU among users.

Contradicting the results from Yuduang et al. [10], intention was not seen to be the highest significant factor, rather habit and hedonic motivation, followed by perceived risk and understanding of COVID-19 pandemic were. These factors were ranked second, third, fourth and sixth in their study. This study clearly showed how the nonlinear relationship due to the presence of a full mediator affects the findings from SEM. The utilization of MLA, specifically ANN, could eliminate these barriers since the presence of hidden layers would help calculate complex models despite the presence of mediators [43]. In studies with more mediators, more hidden layers could be utilized to enhance the accuracy of the findings [42].

### 5.3. Practical and Theorertical Implications

The MorChana COVID-19 contact tracing application was seen to be highly beneficial due to its ease of use, people’s understanding of the benefit it may bring, and its help in avoiding drastic effects on a person due to the avoidance and real-time update of the COVID-19 danger. This could therefore be capitalized on by developers upon creating a health-related application. The findings and the results could be applied for consideration upon creating a similar technology. On the other hand, the framework integrated both UTAUT2 and PMT to evaluate the health-related mobile application. The results showed positive and highly valid constructs. Thus, the items used in this study could be applied and extended in evaluating other health-related mobile applications or technology.

In addition, the methodology and tools considered in this study showed promising results. MLA has been seen to be the trend when it comes to evaluating human factors and behaviors. Most studies [21,22,23,42,43] have integrated MLA and compared it with other statistical and multivariate tools such as structural equation modeling (SEM). With the presented methodology, the sole utility of MLA such as RFC and ANN could be used for evaluating human factors and behavior, even without SEM. Therefore, other studies may apply and extend the utility of RFC and ANN, and even other MLAs to evaluate human behavior studies worldwide.

With the trend of utilizing the MLA ensemble relating to human factors and behavior, this study was able to validate its application through the perceived usability of technology. Though several studies have debated its application and usage as a tool, other studies have been published that considered and utilized the MLA ensemble for human factors and human behavior. Challenges regarding the applicability have risen due to the debate of its predictive power. Thus, our study may contribute towards the small step in further justifying the use and applicability of MLA for predictive and classification purposes with regard to behavioral studies.

### 5.4. Limitations

Despite the significant results and discussion presented, several limitations were still seen with this study. First, this study only considered RFC and ANN as machine learning algorithms. Though both present a model with high accuracy, other MLAs are still highly reliable, with more complex calculations. It is suggested to consider using the Support Vector Machine or Naïve Bayes Algorithm to create a nonlinear relationship among the factors considered. Second, only factors affecting a dependent variable were analyzed in this study. It is suggested to consider clustering the indicators to highlight key features of constructs affecting actual usage among different factors. Lastly, collecting responses among different age groups, educational attainment, and experience could be considered. Clustering such as fuzzy C-means may be done to determine the demographic profile among the MorChana COVID-19 contact tracing application users. This could then be beneficial to developers to have a more generalized device for different age groups and people of different status to use.

## 6. Conclusions

It has been two years since the COVID-19 pandemic started, but until the present, several countries had made little progress in emerging from the pandemic. With the constant mutation and development of COVID-19 variants, the need to reduce the spread should be explored. The development of COVID-19 contact tracing applications has been seen to help mitigate the spread [8]. However, limited studies have explored the different mobile applications’ actual usage, specifically the MorChana COVID-19 contact tracing mobile application from Thailand.

This study aimed to predict and classify factors affecting the actual usage of the Morchana COVID-19 contact tracing mobile application. In addition, the utilization of a machine learning algorithm (MLA) was considered to prove how it could be used to assess factors affecting human behavior following the suggestion and considered limitation of the traditional multivariate analysis such as SEM among several studies [19,20,92,93,94]. MLA tools such as RFC and ANN have been utilized to assess significant factors and importance affecting the actual use of MorChana. The integrated framework of PMT and UTAUT2 was evaluated with 907 valid responses collected via convenience sampling. Factors such as Performance Expectancy (PE), Effort Expectancy (EE), Social Influence (SI), Facilitating Conditions (FC), Hedonic Motivation (HM), Habit (HB), Perceived Risk (PCR), Self-Efficacy (SE), Privacy (PR), Trust (PT), Understanding COVID-19 (U), Intention to Use (IU), and Actual Use (AU) were evaluated simultaneously in this study.

From the validated results, it was seen that HM and FC were important factors that would lead to very high actual use. In addition, HB and U were significant factors that would lead to the high actual use of the MorChana COVID-19 contact tracing mobile application. It was seen that when people understand the impact and causes of the COVID-19 aftermath, its severity, and a way to reduce it, it would lead to the actual usage of a system that would cater to the advantages of health-related systems or technology. Moreover, when users find that there is an ease and health benefit upon using a health-related application, a higher IU would be seen, which would lead to high AU. In addition, the constant usage would lead to the development of a habit and with the enjoyment seen from using the application, HM would be seen, which would affect AU.

It is suggested that the developers and stakeholders take advantage of the results and findings of this study to creating health-related mobile applications. This will encourage people to attain higher AU, which would be beneficial for the users and developers. In addition, profitability is suggested for future researchers to consider. The methodology utilized in this study could be applied and extended to consider MLA for human behavioral studies aside from the multivariate tools available. The advancement of the analysis tools is one of the trends to which this study contributed to the available works of literature. In addition, the integrated framework utilized in this study showed how it could holistically measure factors affecting intention and actual use of a health-related mobile application. Future studies may consider the integration of PMT and UTAUT2 in evaluating factors affecting the AU of health-related applications, or even developing applications from other countries. Lastly, the findings, methodology, and tools of this study could be applied and extended to assess other systems or technology, even human-behavior-related studies worldwide [95,96].

## Figures and Tables

**Table 1:** Literature Review.

Author	COVID-19 Contact Tracing/Country	Method	Purpose	Findings
Altman et al. [25]	France, Germany, Italy, United Kingdom, and United States	Multivariate regression analysis	Investigated the user acceptability of a contact-tracing app in five countries hit by the pandemic.	-strong support for the app under both regimes, in all countries, across all subgroups of the population, and irrespective of regional-level COVID-19 mortality rates -concerns about cybersecurity and privacy, together with a lack of trust in the government, are the main barriers to adoption
Shahroz et al. [26]	General COVID-19 contact tracing.	Comprehensive review analysis	To conduct a comprehensive analysis of digital contact tracing and its supporting IoT framework.	-Different countries and applications have made different trade-offs and have therefore experienced different amounts of success in effectively combating COVID-19. - It was noted that while digital contact tracing apps have their strengths, it is not a panacea. Merging techniques with mobile contact tracing should be considered since others do not attempt to highly utilize it.
Nazayer et al. [27]	General COVID-19 contact tracing.	Comprehensive review analysis	Examine design decisions related to COVID-19 contact-tracing applications and the implications of these decisions.	-Based on the used technology and the software architecture, different contact-tracing applications offer different possible trade-offs that should be taken into account based on government’s objectives on contact tracing.
Zhang et al. [28]	COVIDSafe (Australia); BeAware (Bahrain); CoronApp (Chile); GH COVID-19 Tracker (Ghana); Rakning C-19 (Iceland); NZ COVID Tracer (New Zealand); TraceTogether (Singapore)	Readability analysis	Examined the readability of privacy policies of contact-tracings apps	-Explanation used in the privacy policies of these apps require considerably higher than the reading ability of the average individual.
Seto et al. [29]	General COVID-19 contact tracing.	Comprehensive review analysis	Analyzed how characteristics of contract tracing and exposure notification apps contribute to the perceived levels of privacy awarded to citizens and how this affects an app’s effectiveness	-Striking the right balance between privacy and effectiveness requires careful consideration, especially as the urgency to reduce transmission of the virus evolves based on fluctuating case numbers and vaccination efforts. -contact tracing and exposure notification app will depend on factors including whether it is voluntary, the underlying technology, and degree of data centralization, translation of those important safeguards into a user’s perception of privacy will occur within the context of the norms and values of their country.
Vogt et al. [30]	COVIDSafe (New South Wales, Australia)	Prospective study	To assess the effectiveness of the app to detect close contacts and prevent public exposure events, and its usefulness during the contact identification and risk assessment process	-COVIDSafe generated a substantial additional perceived workload for public health staff and was not considered useful. - COVIDSafe was not sufficiently effective to make a meaningful contribution to the COVID-19 response in Australia’s most populous state over a 6-month period.
Oyibo et al. [31]	General COVID-19 contact tracing.	Comprehensive review analysis	To uncover the key factors that facilitate or militate against the adoption of CTAs, which researchers, designers and other stakeholders should focus on in future iterations to increase their adoption and effectiveness	-Priority to privacy protection through minimal data collection and transparency, improving contact tracing benefits (personal and social), and fostering trust through laudable gestures such as delegating contact tracing to public health authorities, making source code publicly available and stating who will access user data, when, how, and what it will be used for. -the results suggest that data security and tailored persuasive design, involving reward, self-monitoring, and social-location monitoring features, have the potential of improving CTA adoption. -future designs of CTAs to improve their motivational appeal, adoption, and the user experience should be considered.
Owusu [32]	General COVID-19 contact tracing.	Comprehensive review analysis	To review and recommend evaluation of COVID-19 contact tracing applications.	-as with any new system, strong regulatory frameworks are necessary to ensure that individual information is not used for surveillance purposes, and user privacy will be maintained. Having safeguarded this, perhaps the global health community will witness the beginning of a new era of implementing mass health programs through the medium of digital technology.
Zetterholm et al. [33]	General COVID-19 contact tracing.	Comprehensive review analysis	To describe the current knowledge about public acceptance of CTAs and identify individual perspectives, which are essential to consider concerning CTA acceptance and adoption	-Public acceptance varies across national cultures and sociodemographic strata. Lower acceptance among people who are mistrusting, socially disadvantaged, or those with low technical skills suggest a risk that CTAs may amplify existing inequities. -Regarding determinants of acceptance, eight themes emerged, covering both attitudes and behavioral perspectives that can influence acceptance, including trust, privacy concerns, social responsibility, perceived health threat, experience of and access to technologies, performance expectancy and perceived benefits, and understanding.
Gupta et al. [34]	General COVID-19 contact tracing.	Structured research review-based framework	-To review various components of the framework that are related to technological working, design architecture, and feature analysis of the applications, along with the analysis of the acceptance of such applications worldwide. -Components focusing on the security features and analysis of these applications based on Data Privacy, Security Vetting, and different attacks have been included in the research framework.	This study acted as a guide for the users researching contact tracings applications using the proposed four-layered framework for their app assessment.
Kostka and Habich-Sobiegalla [35]	China, Germany, and the United States.	Linear Regression	Analyze public perceptions toward CTAs and the factors that drive CTA acceptance in China, Germany, and the United States.	-Citizens are willing to accept digital contact tracing despite concerns about privacy infringement and government surveillance, as long as the apps are perceived as effective in lowering infection rates and providing health information. -A high citizen adoption rate is necessary for CTAs to be effective, but CTAs are only effective if adoption rates are high.
Vogt et al. [36]	General COVID-19 contact tracing.	Systematic Review	To present a protocol for a systematic review of the main factors, including facilitators and barriers, that influence the adoption of contact tracing apps.	Focus on the principal adoption factors necessary to create better and more effective contact tracing apps.
Albastaki [37]	Bahrain	Structural Equation Modeling	Considered human task performance measures, technology acceptance model, and system usability scale to evaluate perceived usability.	Usability in general rather than factors affecting behavior for utility was the most significant factor.
Storni et al. [38]	General COVID-19 contact tracing.	Systematic Review	Evaluated frameworks used to assess the usability of contact tracing applications.	Highlighted that usability pillars such as satisfaction, availability, accessibility, flexibility, effectiveness, interaction, and ongoing application evaluations were factors that needed to be considered.
Winter et al. [39]	Germany	Eye-tracking and a retrospective think-aloud approach	Evaluated eye tracking and think aloud approach for perceived usability.	They have only concluded that the application is promising and privacy policy has the most significant factor affecting its usability.
Blacklow et al. [40]	United States.	Thematic Analysis	To evaluate the usability using thematic analysis.	Lacked a lot of analysis to be generalized among other contact tracing mobile applications.
Chuenyindee et al. [8]	ThaiChana (Thailand)	Structural Equation Modeling	To determine factors affecting the perceived usability of Thai Chana by integrating protection motivation theory, the extended technology acceptance model, and the system usability scale.	-understanding of COVID-19 has significant effects on perceived severity and perceived vulnerability, which subsequently leads to perceived usefulness. In addition, perceived usefulness and perceived ease of use have significant direct effects on attitude, which subsequently leads to the intention to use, actual use, and perceived usability.
Yuduang et al. [10]	MorChana (Thailand)	Structural Equation Modeling	To evaluate the factors affecting the actual usage of the MorChana mobile application.	Intention was seen to be the most significant indicator. Further evaluation was recommended due to limitations set by the method utilized.
Bente et al. [41]	CoronaMedler application (Netherlands)	Think-aloud usability test and eye-tracking.	To assess usability of the COVID-19 contact tracing application in Netherlands.	-Easy to use, but several demographics found it difficult to interpret, had low trust in privacy, and has been evaluated as underprepared. -Presented ungeneralizable results.
This study	ThaiChana (Thailand)	Machine Learning Ensemble	To evaluate factors affecting perceived usability of the COVID-19 contact tracing application.	Comapred to the results obtained from Yuduang et al. [10], this study highlighted how other factors that were affected from the mediating effect from structural equation modeling was highly significant. In addition, a comparison to other studies, usability of contact tracing, and application has been discussed.

**Table 2:** Validity and Multicollinearity.

Construct	Cronbach’s Alpha	VIF
HB	0.904	4.576
HM	0.889	4.312
PR	0.951	3.628
FC	0.856	3.111
U	0.851	1.341
PE	0.753	2.166
SEF	0.875	2.160
IU	0.802	3.084
TR	0.855	2.605
EE	0.885	2.071
PCR	0.705	1.161
SI	0.946	3.323

**Table 3:** Decision Tree Mean Accuracy (Depth = 5).

Category	60:40	70:30	80:20	90:10
**Random**				
**Gini**	84.00	80.00	83.20	83.60
**Std. Dev**	4.301	4.743	4.438	6.542
**Precision**	81.40	77.00	82.80	83.20
**Recall**	75.40	73.20	80.00	72.80
**Entropy**	85.00	80.60	86.00	84.80
**Std. Dev**	1.732	2.510	1.225	7.430
**Precision**	84.80	81.00	85.80	79.80
**Recall**	83.40	74.20	81.80	63.80
**Best**				
**Gini**	88.80	83.80	93.00	91.00
**Std. Dev**	0.447	1.095	0.000	0.000
**Precision**	86.80	82.80	92.80	91.40
**Recall**	84.60	81.40	90.60	89.80
**Entropy**	83.20	84.60	89.40	92.00
**Std. Dev**	1.095	0.894	0.547	0.000
**Precision**	83.00	82.80	89.80	90.00
**Recall**	83.00	83.80	89.60	91.00

**Table 4:** Pearson’s R Correlation.

Latent	HB	HM	PCR	FC	U	PE	SEF	IU	TR	EE	PR	SI
HM	0.866											
PCR	0.344	0.329										
FC	0.787	0.777	0.305									
U	0.479	0.487	0.193	0.417								
PE	0.694	0.681	0.309	0.625	0.381							
SEF	0.693	0.678	0.247	0.612	0.399	0.567						
IU	0.787	0.764	0.306	0.701	0.418	0.620	0.592					
TR	0.745	0.740	0.269	0.657	0.385	0.607	0.594	0.678				
EE	0.692	0.657	0.274	0.607	0.393	0.534	0.579	0.602	0.616			
PR	0.812	0.789	0.337	0.719	0.446	0.639	0.642	0.726	0.691	0.616		
SI	0.800	0.777	0.310	0.708	0.426	0.639	0.625	0.715	0.664	0.617	0.749	
AU	0.847	0.831	0.602	0.760	0.761	0.702	0.675	0.568	0.363	0.432	0.460	0.333

## Data Availability

The data presented in this study are available on request from the corresponding author.

## References

[1] Ong A.K., Prasetyo Y.T., Picazo K.L., Salvador K.A., Miraja B.A., Kurata Y.B., Chuenyindee T., Nadlifatin R., Redi A.A., Young M.N. (2021). Gym-goers preference analysis of fitness centers during the COVID-19 pandemic: A conjoint analysis approach for business sustainability. Sustainability.

[2] Issac A., Radhakrishnan R.V., Vijay V., Stephen S., Krishnan N., Jacob J., Jose S., Azhar S., Nair A.S. (2021). An examination of Thailand’s health care system and strategies during the management of the COVID-19 pandemic. J. Glob. Health.

[3] Thailand Launches Mor Chana Mobile App to Enhance Contact Tracing Efforts to Help Stop the Spread of COVID-19. https://www.cattelecom.com/cat/content/3754/222/Thailand+launches+Mor+Chana+mobile+app+to+enhance+?lang=en_EN.

[4] Viwattanakulvanid P. (2021). Ten commonly asked questions about COVID-19 and lessons learned from Thailand. J. Health Res..

[5] Reinecke K., Bernstein A. (2011). Improving performance, perceived usability, and aesthetics with culturally adaptive user interfaces. ACM Trans. Comput.-Hum. Interact. (TOCHI).

[6] Hidayat-ur-Rehman I., Ahmad A., Ahmed M., Alam A. (2021). Mobile applications to fight against COVID-19 pandemic: The Case of Saudi Arabia. Tem J.-Technol. Educ. Manag. Inform..

[7] Zhou S.L., Jia X., Skinner S.P., Yang W., Claude I. (2021). Lessons on mobile apps for COVID-19 from China. J. Saf. Sci. Resil..

[8] Chuenyindee T., Ong A.K., Prasetyo Y.T., Persada S.F., Nadlifatin R., Sittiwatethanasiri T. (2022). Factors affecting the perceived usability of the COVID-19 contact-tracing application “Thai chana” during the early COVID-19 omicron period. Int. J. Environ. Res. Public Health.

[9] Venkatesh V., Thong J.Y., Xu X. (2012). Consumer acceptance and use of information technology: Extending the unified theory of acceptance and use of technology. MIS Q..

[10] Yuduang N., Ong A.K., Prasetyo Y.T., Chuenyindee T., Kusonwattana P., Limpasart W., Sittiwatethanasiri T., Gumasing M.J., German J.D., Nadlifatin R. (2022). Factors influencing the perceived effectiveness of COVID-19 risk assessment mobile application “Morchana” in Thailand: Utaut2 approach. Int. J. Environ. Res. Public Health.

[11] Alam M.Z., Hu W., Kaium M.A., Hoque M.R., Alam M.M.D. (2020). Understanding the determinants of mHealth apps adoption in Bangladesh: A SEM-Neural network approach. Technol. Soc..

[12] Badran M.F. (2019). eHealth in Egypt: The demand-side perspective of implementing electronic health records. Telecommun. Policy.

[13] Mamra A., Sibghatullah A.S., Ananta G.P., Alazzam M.B., Ahmed Y.H., Doheir M. (2017). A proposed framework to investigate the user acceptance of personal health records in Malaysia using UTAUT2 and PMT. Int. J. Adv. Comput. Sci. Appl..

[14] Prasetyo Y.T., Castillo A.M., Salonga L.J., Sia J.A., Seneta J.A. (2020). Factors affecting perceived effectiveness of COVID-19 prevention measures among Filipinos during enhanced community quarantine in Luzon, Philippines: Integrating Protection Motivation Theory and extended Theory of Planned Behavior. Int. J. Infect. Dis..

[15] Ong A.K., Prasetyo Y.T., Salazar J.M., Erfe J.J., Abella A.A., Young M.N., Chuenyindee T., Nadlifatin R., Ngurah Perwira Redi A.A. (2021). Investigating the acceptance of the reopening Bataan Nuclear Power Plant: Integrating Protection Motivation Theory and extended theory of planned behavior. Nucl. Eng. Technol..

[16] van Bavel R., Rodríguez-Priego N., Vila J., Briggs P. (2019). Using protection motivation theory in the design of nudges to improve online security behavior. Int. J. Hum.-Comput. Stud..

[17] Mousavi R., Chen R., Kim D.J., Chen K. (2020). Effectiveness of privacy assurance mechanisms in users’ privacy protection on social networking sites from the perspective of protection motivation theory. Decis. Support Syst..

[18] Yu C.-W., Chao C.-M., Chang C.-F., Chen R.-J., Chen P.-C., Liu Y.-X. (2021). Exploring Behavioral Intention to Use a Mobile Health Education Website: An Extension of the UTAUT 2 Model. SAGE Open.

[19] Fan Y., Chen J., Shirkey G., John R., Wu S.R., Park H., Shao C. (2016). Applications of structural equation modeling (SEM) in ecological studies: An updated review. Ecol. Processes.

[20] Woody E. (2011). An SEM perspective on evaluating mediation: What every clinical researcher needs to know. J. Exp. Psychopathol..

[21] Chen J., Li Q., Wang H., Deng M. (2020). A machine learning ensemble approach based on random forest and radial basis function neural network for risk evaluation of regional flood disaster: A case study of the Yangtze River Delta, China. Int. J. Environ. Res. Public Health.

[22] Milani L., Grumi S., Camisasca E., Miragoli S., Traficante D., Di Blasio P. (2020). Familial risk and protective factors affecting CPS Professionals’ Child Removal Decision: A decision tree analysis study. Child. Youth Serv. Rev..

[23] Al-Mashraie M., Chung S.H., Jeon H.W. (2020). Customer switching behavior analysis in the telecommunication industry via push-pull-mooring framework: A machine learning approach. Comput. Ind. Eng..

[24] Jamshidi M., Roshani S., Daneshfar F., Lalbakhsh A., Roshani S., Parandin F., Malek Z., Talla J., Peroutka Z., Jamshidi A. (2022). Hybrid deep learning techniques for predicting complex phenomena: A review on COVID-19. AI.

[25] Altmann S., Milsom L., Zillessen H., Blasone R., Gerdon F., Bach R., Kreuter F., Nosenzo D., Toussaert S., Abeler J. (2020). Acceptability of APP-based contact tracing for COVID-19: Cross-country survey study. JMIR Mhealth Uhealth.

[26] Shahroz M., Ahmad F., Younis M.S., Ahmad N., Kamel Boulos M.N., Vinuesa R., Qadir J. (2021). COVID-19 digital contact tracing applications and techniques: A review post initial deployments. Transp. Eng..

[27] Nazayer M., Madanian S., Mirza F. (2021). Contact-Tracing Applications: A review of Technologies. BMJ Innov..

[28] Zhang M., Chow A., Smith H. (2020). COVID-19 contact-tracing apps: Analysis of the readability of privacy policies. J. Med. Internet Res..

[29] Seto E., Challa P., Ware P. (2021). Adoption of COVID-19 contact tracing apps: A balance between privacy and effectiveness. J. Med. Internet Res..

[30] Vogt F., Haire B., Selvey L., Kaldor J. (2021). Effectiveness of digital contact tracing for COVID-19 in New South Wales, Australia. Lancet Public Health.

[31] Oyibo K., Sahu K.S., Oetomo A., Morita P.P. (2021). Factors influencing the adoption of Contact Tracing Applications: Protocol for a systematic review. JMIR Res. Protoc..

[32] Owusu P.N. (2020). Digital Technology applications for contact tracing: The new promise for COVID-19 and beyond?. Glob. Health Res. Policy.

[33] Zetterholm M.V., Lin Y., Jokela P. (2021). Digital contact tracing applications during COVID-19: A scoping review about public acceptance. Informatics.

[34] Gupta R., Pandey G., Chaudhary P., Pal S.K. (2021). Technological and analytical review of contact tracing apps for COVID-19 management. J. Locat. Based Serv..

[35] Kostka G., Habich-Sobiegalla S. (2022). In times of crisis: Public perceptions toward COVID-19 contact tracing apps in China, Germany, and the United States. New Media Soc..

[36] Vogt F., Kurup K., Mussleman P., Habrun C., Crowe M., Woodward A., Jaramillo-Gutierrez G., Kaldor J., Vong S., Del Rio Vilas V.J. (2022). Contact tracing indicators for COVID-19: Rapid scoping review and conceptual framework. PLoS ONE.

[37] Albastaki Y. (2022). Assessing the perceived usability of an intelligent contact tracing app to prevent the spread of COVID-19 using Sus and Tam: Be aware bahrain. J. Decis. Syst..

[38] Storni C., Tsvyatkova D., Richardson I., Buckley J., Abbas M., Beecham S., Chochlov M., Fitzgerald B., Glynn L., Johnson K. Toward a compare and contrast framework for COVID-19 contact tracing mobile applications: A look at usability. Proceedings of the 14th International Joint Conference on Biomedical Engineering Systems and Technologies.

[39] Winter M., Baumeister H., Frick U., Tallon M., Reichert M., Pryss R. Exploring the usability of the German COVID-19 contact tracing app in a combined eye tracking and retrospective think Aloud Study. Proceedings of the 2021 43rd Annual International Conference of the IEEE Engineering in Medicine & Biology Society (EMBC).

[40] Blacklow S.O., Lisker S., Ng M.Y., Sarkar U., Lyles C. (2021). Usability, inclusivity, and content evaluation of COVID-19 contact tracing apps in the United States. J. Am. Med. Inform. Assoc..

[41] Bente B.E., van’t Klooster J.W., Schreijer M.A., Berkemeier L., van Gend J.E., Slijkhuis P.J., Kelders S.M., van Gemert-Pijnen J.E. (2021). The Dutch COVID-19 contact tracing app (the coronamelder): Usability study. JMIR Form. Res..

[42] Ong A.K., Chuenyindee T., Prasetyo Y.T., Nadlifatin R., Persada S.F., Gumasing M.J., German J.D., Robas K.P., Young M.N., Sittiwatethanasiri T. (2022). Utilization of random forest and deep learning neural network for predicting factors affecting perceived usability of a COVID-19 contact tracing mobile application in Thailand “Thaichana”. Int. J. Environ. Res. Public Health.

[43] Ong A.K., Prasetyo Y.T., Velasco K.E., Abad E.D., Buencille A.L., Estorninos E.M., Cahigas M.M., Chuenyindee T., Persada S.F., Nadlifatin R. (2022). Utilization of random forest classifier and artificial neural network for predicting the acceptance of reopening decommissioned nuclear power plant. Ann. Nucl. Energy.

[44] Jamshidi M., Lalbakhsh A., Talla J., Peroutka Z., Hadjilooei F., Lalbakhsh P., Jamshidi M., Spada L.L., Mirmozafari M., Dehghani M. (2020). Artificial Intelligence and COVID-19: Deep learning approaches for diagnosis and treatment. IEEE Access.

[45] Daube C., Xu T., Zhan J., Webb A., Ince R.A.A., Garrod O.G.B., Schyns P.G. (2021). Grounding deep neural network pre-dictions of human categorization behavior in understandable functional features: The case of Face Identity. Patterns.

[46] Jamshidi M.B., Lalbakhsh A., Talla J., Peroutka Z., Roshani S., Matousek V., Roshani S., Mirmozafari M., Malek Z., La Spada L. (2021). Deep learning techniques and COVID-19 drug discovery: Fundamentals, state-of-the-art and Future Directions. Stud. Syst. Decis. Control..

[47] Yariyan P., Zabihi H., Wolf I.D., Karami M., Amiriyan S. (2020). Earthquake risk assessment using an integrated Fuzzy Analytic Hierarchy Process with Artificial Neural Networks based on GIS: A case study of Sanandaj in Iran. Int. J. Disaster Risk Reduct..

[48] Oktarina R., Bahagia S.N., Diawati L., Pribadi K.S. (2020). Artificial neural network for predicting earthquake casualties and damages in Indonesia. IOP Conf. Series Earth Environ. Sci..

[49] Aggarwal C.C. (2019). Neural Networks and Deep Learning: A Textbook.

[50] Kheirollahpour M.M., Danaee M.M., Merican A.F., Shariff A.A. (2020). Prediction of the influential factors on eating behaviors: A hybrid model of structural equation modelling-artificial neural networks. Sci. World J..

[51] Featherman M.S., Pavlou P.A. (2003). Predicting e-services adoption: A perceived risk facets perspective. Int. J. Hum.-Comput. Stud..

[52] Thakur R. (2018). The role of self-efficacy and customer satisfaction in driving loyalty to the mobile shopping application. Int. J. Retail. Distrib. Manag..

[53] Kim H., Suh E.E. (2018). The effects of an interactive nursing skills mobile application on nursing students’ knowledge, self-efficacy, and skills performance: A randomized controlled trial. Asian Nurs. Res..

[54] Ezzaouia I., Bulchand-Gidumal J. (2021). A Model to Predict Users’ Intentions to Adopt Contact-Tracing Apps for Prevention from COVID-19. Information and Communication Technologies in Tourism 2021.

[55] Nikolopoulou K., Gialamas V., Lavidas K. (2021). Habit, hedonic motivation, performance expectancy and technological pedagogical knowledge affect teachers’ intention to use mobile internet. Comput. Educ. Open.

[56] Chen W., Chan T.W., Wong L.H., Looi C.K., Liao C.C., Cheng H.N., Wong S.L., Mason J., So H.-J., Murthy S. (2020). IDC theory: Habit and the habit loop. Res. Pract. Technol. Enhanc. Learn..

[57] Amoroso D., Lim R., Roman F.L. (2021). Developing and Testing a Smartphone Dependency Scale Assessing Addiction Risk. Int. J. Risk Conting. Manag..

[58] Cao Q., Niu X. (2019). Integrating context-awareness and UTAUT to explain Alipay user adoption. Int. J. Ind. Ergon..

[59] Martins C., Oliveira T., Popovič A. (2014). Understanding the Internet banking adoption: A unified theory of acceptance and use of technology and perceived risk application. Int. J. Inf. Manag..

[60] Mingxing S., Jing F., Yafang L. An empirical study on consumer acceptance of mobile payment based on the perceived risk and trust. Proceedings of the 2014 International Conference on Cyber-Enabled Distributed Computing and Knowledge Discovery.

[61] Lallmahomed M.Z., Lallmahomed N., Lallmahomed G.M. (2017). Factors influencing the adoption of e-Government services in Mauritius. Telemat. Inform..

[62] Walrave M., Waeterloos C., Ponnet K. (2021). Ready or not for contact tracing? Investigating the adoption intention of COVID-19 contact-tracing technology using an extended unified theory of acceptance and use of technology model. Cyberpsychol. Behav. Soc. Netw..

[63] Wu J., Du H. (2012). Toward a better understanding of behavioral intention and system usage constructs. Eur. J. Inf. Syst..

[64] Dehghani M. (2018). Exploring the motivational factors on continuous usage intention of smartwatches among actual users. Behav. Inf. Technol..

[65] Huang C.-Y., Yang M.-C. (2020). Empirical investigation of factors influencing consumer intention to use an artificial intelligence-powered mobile application for weight loss and health management. Telemed. e-Health.

[66] Hair J.F., Black W.C., Babin B.J., Anderson R.E. (2010). Multivariate Data Analysis.

[67] Podsakoff P.M., MacKenzie S.B., Lee J.-Y., Podsakoff N.P. (2003). Common method biases in behavioral research: A critical review of the literature and recommended remedies. J. Appl. Psychol..

[68] Akoglu H. (2018). User’s Guide to Correlation Coefficients. Turk. J. Emerg. Med..

[69] Yang W., Zhou S. (2020). Using decision tree analysis to identify the determinants of residents’ CO_2_ emissions from different types of trips: A case study of guangzhou, China. J. Clean. Prod..

[70] Pradhan B., Lee S. (2010). Landslide susceptibility assessment and factor effect analysis: Backpropagation artificial neural networks and their comparison with frequency ratio and bivariate logistic regression modelling. Environ. Model. Softw..

[71] Walczak S., Cerpa N. (2003). Artificial Neural Networks. Encyclopedia of Physical Science and Technology.

[72] Wang X., Ren H., Wang A. (2022). Smish: A novel activation function for deep learning methods. Electronics.

[73] Lin G., Shen W. (2018). Research on convolutional neural network based on improved relu piecewise activation function. Procedia Comput. Sci..

[74] Kalinić Z., Marinković V., Kalinić L., Liébana-Cabanillas F. (2021). Neural network modeling of consumer satisfaction in Mobile Commerce: An empirical analysis. Expert Syst. Appl..

[75] Sharma S., Sharma A., Athaiya A. (2020). Activation Functions in Neural Network. Int. J. Eng. Appl. Sci. Technol..

[76] Elfwing S., Uchibe E., Doya K. (2018). Sigmoid-weighted linear units for neural network function approximation in reinforcement learning. Neural Netw..

[77] Gudivada V.N., Rao C.R. (2018). Computational analysis and understanding of natural languages: Principles, methods and applications. Handbook of Statistics.

[78] Jang H.-S., Xing S. (2020). A model to predict ammonia emission using a modified genetic artificial neural network: Analyzing Cement mixed with fly ash from a coal-fired power plant. Constr. Build. Mater..

[79] Yousefzadeh M., Hosseini S.A., Farnaghi M. (2021). Spatiotemporally explicit earthquake prediction using Deep Neural Network. Soil Dyn. Earthq. Eng..

[80] Costarelli D., Spigler R. (2013). Multivariate neural network operators with sigmoidal activation functions. Neural Netw..

[81] Liébana-Cabanillas F., Marinković V., Kalinić Z. (2017). A sem-neural network approach for predicting antecedents of M-Commerce Acceptance. Int. J. Inf. Manag..

[82] Maida A.S. (2016). Cognitive computing and neural networks. Handbook of Statistics.

[83] Abiodun O.I., Jantan A., Omolara A.E., Dada K.V., Mohamed N.A., Arshad H. (2018). State-of-the-art in artificial neural network applications: A survey. Heliyon.

[84] Wu P., Zhang R., Zhu X., Liu M. (2022). Factors Influencing Continued Usage Behavior on Mobile Health Applications. Healthcare.

[85] Palau-Saumell R., Forgas-Coll S., Sánchez-García J., Robres E. (2019). User acceptance of mobile apps for restaurants: An expanded and extended UTAUT-2. Sustainability.

[86] Shemesh T., Barnoy S. (2020). Assessment of the intention to use mobile health applications using a technology acceptance model in an Israeli adult population. Telemed. e-Health.

[87] Tuman M., Moyer A. (2019). Health intentions and behaviors of health app owners: A cross-sectional study. Psychol. Health Med..

[88] Wang C., Tee M., Roy A.E., Fardin M.A., Srichokchatchawan W., Habib H.A., Tran B.X., Hussain S., Hoang M.T., Le X.T. (2021). The impact of COVID-19 pandemic on physical and mental health of Asians: A study of seven middle-income countries in Asia. PLoS ONE.

[89] Caldwell J., de Lara-Tuprio E., Teng T.R., Estuar R.J., Sarmiento R.F.R., Abayawardana M., Leong R.N.F., Gray R.T., Wood J., McBryde E.S. (2021). Understanding COVID-19 dynamics and the effects of interventions in the Philippines: A mathematical modelling study. Lancet Reg. Health.

[90] Chao C.-M. (2019). Factors determining the behavioral intention to use mobile learning: An application and extension of the UTAUT model. Front. Psychol..

[91] Tomczyk S., Barth S., Schmidt S., Muehlan H. (2021). Utilizing health behavior change and technology acceptance models to predict the adoption of COVID-19 contact tracing apps: Cross-sectional Survey Study. J. Med. Internet Res..

[92] German J.D., Redi A.A., Prasetyo Y.T., Persada S.F., Ong A.K., Young M.N., Nadlifatin R. (2022). Choosing a package carrier during COVID-19 pandemic: An integration of pro-environmental planned behavior (PEPB) theory and Service Quality (SERVQUAL). J. Clean. Prod..

[93] Kurata Y.B., Prasetyo Y.T., Ong A.K., Nadlifatin R., Chuenyindee T. (2022). Factors affecting perceived effectiveness of typhoon vamco (Ulysses) flood disaster response among Filipinos in Luzon, Philippines: An integration of protection motivation theory and extended theory of planned behavior. Int. J. Disaster Risk Reduct..

[94] Gumasing M.J., Prasetyo Y.T., Ong A.K., Nadlifatin R. (2022). Determination of factors affecting the response efficacy of Filipinos under Typhoon Conson 2021 (jolina): An extended protection motivation theory approach. Int. J. Disaster Risk Reduct..

[95] Garrett P.M., Wang Y.-W., White J.P., Kashima Y., Dennis S., Yang C.-T. (2022). High acceptance of COVID-19 Tracing Technologies in Taiwan: A nationally representative survey analysis. Int. J. Environ. Res. Public Health.

[96] Abella A.A., Prasetyo Y.T., Young M.N., Nadlifatin R., Persada S.F., Perwira Redi A.A., Chuenyindee T. (2022). The effect of positive reinforcement of behavioral-based safety on safety participation in Philippine coal-fired power plant workers: A partial least square structural equation modeling (PLS-SEM) approach. Int. J. Occup. Saf. Ergon..

